# A review of national action plans on antimicrobial resistance: strengths and weaknesses

**DOI:** 10.1186/s13756-022-01130-x

**Published:** 2022-06-23

**Authors:** Angela Willemsen, Simon Reid, Yibeltal Assefa

**Affiliations:** School of Public Health, Faculty of Medicine, The University of Queensland, Herston, QLD Australia

**Keywords:** Antimicrobial resistance, Global action plan, National action plan, Collaboration

## Abstract

**Background:**

The World Health Organization developed the Global Action Plan on Antimicrobial resistance (AMR) as a priority because of the increasing threat posed to human health, animal health and agriculture. Countries around the world have been encouraged to develop their own National Action Plans (NAPs) to help combat AMR.

The objective of this review was to assess the content of the NAPs and determine alignment with the Global Action Plan on Antimicrobial Resistance using a policy analysis approach.

**Body:**

National Action Plans were accessed from the WHO Library and systematically analysed using a policy analysis approach for actors, process, context and content. Information was assessed using a ‘traffic light’ system to determine agreeance with the five WHO Global Action Plans objectives.

A total of 78 NAPs (70 WHO approved, eight not approved) from the five global regions were analysed. National action plans which provided more information regarding the consultative process and the current situation regarding AMR allowed greater insight to capabilities of the country. Despite the availability of guidelines to inform the development of the plans, there were many differences between plans with the content of information provided. High income countries indicated greater progression with objectives achievement while low and middle-income countries presented the need for human and financial resources.

**Conclusion:**

The national action plans provide an overview of activities underway to combat AMR globally. This analysis reveals how disconnected the process has been and how little information is being gathered globally.

**Supplementary Information:**

The online version contains supplementary material available at 10.1186/s13756-022-01130-x.

## Background

The increasing threat of antimicrobial resistance (AMR) continues to impact humans, animals and agriculture. The extent of AMR and the measures countries are taking to address this threat are unknown [1]. Factors contributing to AMR are overuse and misuse of antimicrobials in human health, animal health and agricultural systems [1, 2]. The Global Action Plan (GAP) on Antimicrobial Resistance (AMR) [3] written by the World Health Organisation (WHO) was endorsed at the 68th World Health Assembly in May 2015 [4]. Members of the World Health Assembly recognized that increasing AMR is threatening the future management of current and emerging pathogens. A collaborative One Health approach is vital to engage all stakeholders are working towards a common goal [5].

The WHO GAP on AMR provides countries with a broad framework of how to tackle AMR using five strategic objectives [3]. The strategic objectives are to: (1) improve awareness and understanding of antimicrobial resistance through effective communication, education and training; (2) strengthen the knowledge and evidence base through surveillance and research; (3) reduce the incidence of infection through effective sanitation, hygiene and infection prevention measures; (4) optimize the use of antimicrobial medicines in human and animal health; (5) develop the economic case for sustainable investment that takes account of the needs of all countries and increase investment in new medicines, diagnostic tools, vaccines and other interventions. The WHO GAP for AMR provided guidelines for countries to have AMR national action plans (NAP) in place within two years. Countries were supported to develop and implement their action plans, including the release of a manual in early 2016 [3, 6], developed by WHO, Food and Agriculture Organization of the United Nations and World Organisation for Animal Health.

Despite the availability of the NAPs, the response to AMR is thought to be inadequate. This may be due to factors such as poor alignment of the NAPs with the GAP, inadequate capacity for implementation or poor awareness of the need for addressing AMR. In addition, the availability of a NAP does not ensure that the recommended strategies to combat AMR are enacted or enforced [7]. The importance of, or the inclusion of One Health in the WHO GAP was not distinctly apparent, partly because the term, “One Health is only used three times in the GAP; once in the foreword, then within the consultative process and as part of Objective 1 [3]. This lack of attention fails to instil the need for this collaborative approach from this formative document. The objective of this review was to assess the content of the NAPs and determine their alignment with the GAP, identify policy areas that are absent from NAPs, and identify countries that may need additional support in developing coherent NAPs.

## Methods

We conducted a systematic document review and analysis of NAPs (Additional file 1), which were accessed from the WHO Library of AMR NAPs [8], with NAPs categorized into one of the six WHO Regional Offices: Africa; the Americas; Eastern Mediterranean; Europe; South-East Asia; and Western Pacific.

Each NAP was reviewed manually using an iterative approach to identify frequently used terms and keywords [9]. A word search using Acrobat Reader DC or Microsoft Word was conducted for every National Action Plan using the terms ‘veterinary’, ‘agriculture’, ‘human’, ‘animal’, ‘surveillance’, ‘susceptibility’, ‘gap’, ‘strength’, ‘weakness’, ‘AST’. Each mention of a term was reviewed to ensure the terms were used in the correct context. The term veterinary referred to the provision of veterinary care while agriculture related to farming practices. Data extracted using these search terms and during review of each NAP were included in a Microsoft Excel® (version 2016) worksheet.

We used a policy analysis approach to review the NAPs, extract data and synthesize the evidence. The use of a health policy analysis framework such as the policy triangle framework acknowledged the need to include three factors (actors, context and content) for effective policy development [10]. We reviewed: actors—which sectors (human health, veterinary health, agriculture) were involved in the development of the NAP and if names and substantive positions of contributing individuals were included; context—whether a SWOT (strengths, weaknesses, opportunities and threats) analysis or situation analysis was conducted; and content—if the NAPs addressed the objectives and provided strategies in alignment with those provided in the WHO GAP and if a national antimicrobial surveillance system was in place.

Each NAP was assessed for the inclusion of objectives in line with the five WHO GAP on Antimicrobial Resistance objectives. Alignment of the NAP objectives with the GAP objectives was assessed by comparing objectives directly. Strong alignment (score 2, green traffic light) was determined if the objectives in a country’s NAP were identical to the GAP. The maximum score was 10 for full alignment with all five objectives. Some countries included additional objectives or reworded the GAP objectives. Partial alignment of objectives was determined by identifying similarities in intent. Similarities were highlighted and noted on the NAP objective stating which GAP objective it was most closely aligned with. Partial alignment with the GAP objectives achieved a score of one and coding of yellow. Objectives that were not addressed or absent (i.e. in short, uninformative NAPs) were given a score of zero and coded red. Additional file 2 provides a visual depiction of the attainment of the GAP objectives.

National Action Plans were assessed for multisectoral collaboration between human health, veterinary health and agriculture, a list of contributors to the NAP, and the inclusion of a SWOT analysis. National Action Plans listing contributors and collaboration with representatives from human health, veterinary health and agriculture could achieve a maximum score of eight points, with an additional two points allocated for the inclusion of a SWOT analysis. Plans partially addressing any of these five criteria (collaboration, contributors or SWOT analysis) received a score of one, with zero allocated when information regarding any of these could not be located within the NAP.

**Fig. 1:**
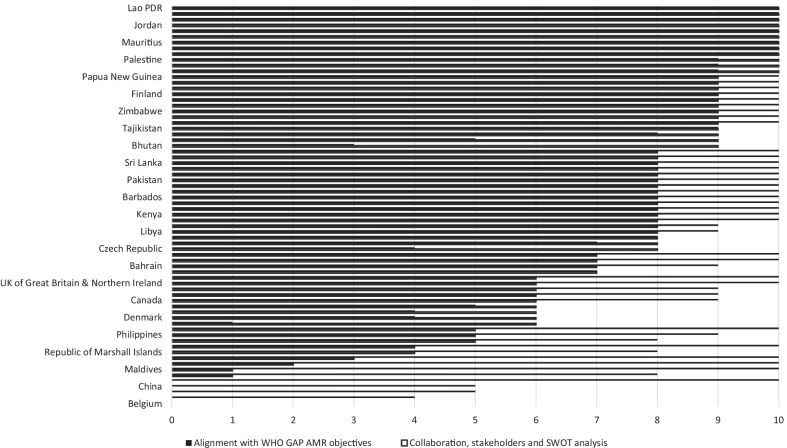
Collaboration and stakeholder involvement/SWOT analysis and global alignment with WHO AMR GAP objectives WHO approved National Action Plans (n = 70) (*Note* not all names of countries visible in figure)

The mean score was calculated for each NAP strategic objective, as depicted in Fig. 1. A score between two and zero (aligned /partially aligned/not aligned or not located) was allocated to WHO approved NAPs and also for WHO approved and non-approved NAPs. Spearman’s correlation coefficient was used to determine if there was any correlation between the five strategic objectives in the NAPs with the GAP. Spearman’s correlation was run to determine the statistical significance of any relationship between the Strategic objectives from the NAPs and the number of stakeholders included in the plan. Analysis was performed using Microsoft Excel at a 95% confidence level.

## Results

The Library of AMR NAPs was reviewed on 3 November 2021 and an additional 10 NAPs had been approved and added to the library. A total of 86 countries have officially approved NAPs (Table 1). Of these, 16 were written in a language other than English so were not reviewed. Only NAPs written in English were analysed (n = 78) (Table 1 and Table 2). This comprises 44% of the 194 countries in the world. A breakdown of the action plans according to their region and whether the NAP is written in English is provided in Table 1. A further eight countries have compiled action plans but have not been officially approved by the WHO (Table 2). These action plans were accessed from the previous database of AMR national action plans which is no longer available. We present the findings of the study using the three components of the policy analysis framework utilized.

**Table 1:** Antimicrobial resistance National Action Plans according to region and country and whether they are written in English (as of 3 November 2021)

Region	Country				
	National action plan in English	Number	National action plan not English	Number	Total number
Africa	Eritrea *Eswatini* Ethiopia *Ghana* Kenya Liberia *Malawi* Mauritius Nigeria Sierra Leone South Africa United Republic of Tanzania *Zambia* *Zimbabwe*	14	Burkina Faso	1	15
Americas	Barbados Canada United States of America	3	Argentina Brazil Costa Rica Paraguay Peru	5	8
Eastern Mediterranean	Afghanistan Bahrain Egypt Iran Iraq Jordan Kingdom of Saudi Arabia Lebanon Libya Oman Pakistan Palestine Sudan United Arab Emirates	14	Morocco Tunisia	2	16
Europe	Belgium Czech Republic Denmark Finland France Germany Ireland Italy Netherlands *Norway* Republic of Serbia *Spain* Sweden Tajikistan The Former Yugoslav Republic of Macedonia Turkmenistan United Kingdom of Great Britain and Northern Ireland	17	Armenia Austria Cyprus Greece Lithuania Republic of Montenegro Portugal	7	24
South-East Asia	Bangladesh Bhutan Democratic Republic of Timor Leste DPR of Korea India Indonesia Maldives Sri Lanka Thailand	9		0	9
Western Pacific	Australia Cambodia China Federated States of Micronesia Fiji *Japan* Lao PDR Mongolia *Nauru* Papua New Guinea Philippines Republic of Marshall Islands *Tuvalu*	13	Republic of Korea	1	14
	Total	70		16	86

(Italicised countries included after WHO NAP Library revisited 2 November 2021)

**Table 2:** National Action Plans not officially approved by World Health Organisation and no longer available on the WHO Library of AMR National Action Plans

Region	Country	Number
Africa	*Zimbabwe*	0
Americas	–	0
Eastern Mediterranean	–	0
Europe	*Norway* *Spain* Switzerland	1
South-East Asia	Malaysia Myanmar Nepal Singapore	4
Western Pacific	Brunei *Japan* New Zealand Vietnam	3
		8

Categorised according to presumed WHO Regional Office. The four italicised countries were moved to officially approved NAPs between April and November 2021 and are included in Table 1

## National action plans and WHO global action plan on antimicrobial resistance

The WHO AMR manual for developing National Action Plans includes mapping of stakeholders, conducting a situational analysis, and developing strategic objectives to develop the NAP for their country. Results are provided for the 70 WHO-approved NAPs and the eight (N = 78) additional non-WHO approved NAPs in Additional file 2. Data is calculated from the traffic light system of the variables in Table 3.

**Table 3:** Mean of each WHO Antimicrobial Resistance strategic objective according to National Action Plans

	Strategic objective 1	Strategic objective 2	Strategic objective 3	Strategic objective 4	Strategic objective 5
WHO approved plans (n = 60)	1.73	1.8	1.65	1.78	1.55
All plans (n = 72)	1.72	1.79	1.5	1.76	1.5

Figure 1 demonstrates global alignment of all 70 WHO approved NAPs with WHO AMR objectives, depicted as solid black columns. The horizontal axis shows the number of allocated points according to alignment with each WHO AMR objective, ranging from zero to 10.The level of collaboration, inclusion of collaborators and inclusion of situational or SWOT analysis is depicted in black outline with each criteria scored from zero to 10 The vertical axis shows the NAPs of the included countries. Additional file 3 provides a visual depiction of all 78 NAPs.

A total of 41 from 70 (59%) approved NAPs countries demonstrated alignment with the WHO GAP strategic objectives. Only one country received zero (Belgium) and one country received a score of one (Bangladesh).

A total of 12 from 70 (17%) approved countries received a score of 10 for recording good multisectoral collaboration, listing of contributors and the inclusion of a SWOT analysis. South Africa, Belgium, FYRoM, Netherlands, China received a score of zero, with all countries from WHO approved NAPs.

A total of nine (13%) approved countries (Ghana, Liberia, Mauritius, Nigeria, United Republic of Tanzania, Jordan, Lebanon, Thailand, Lao PDR received a score of 10 for both alignment with WHO GAP strategic objectives and including information regarding stakeholder collaboration, contributors and SWOT analysis. From a regional perspective, this represented five African countries (56%), two Eastern Mediterranean countries (22%), one South-East Asian country (11%) and one Western Pacific country (11%). No countries from the Americas or Europe were represented. None of the eight non-approved NAPs achieved a complete score in both evaluated areas.

Belgium scored zero for alignment with WHO GAP strategic objectives, however, the English version was only five pages and the more complete Belgian NAP was written in French. Bangladesh scored one point for alignment with WHO GAP strategic objectives as the plan was brief and included brief objectives and activities. Five countries (South Africa, Belgium, Netherlands, The FYRoM, China) scored zero for evaluation of multisectoral collaboration, involvement of stakeholders and SWOT analysis. South Africa included a joint foreword by the Ministers of Health and Agriculture, Forestry and Fisheries. The Belgium, Netherlands and FYRoM NAPs were brief (5–18 pages).

Figure 1 was used to calculate which strategic objective had the greatest alignment across all regions. Objective 2 had the greatest alignment (1.8) with objective 5 showing the lowest level of alignment (1.55). Results are provided in Table 3.

There was a weak, positive correlation between the overall NAP score and the number of stakeholders included in the drafting team (r_s_ = 0.30, n = 72, p < 0.05) (See Fig. 1).

## Regional summaries

### Africa

#### Stakeholders and process

National action plans were written between 2015 (Ethiopia) and 2021 (Eritrea), with most written (n = 9) during 2017. Twelve of the 14 NAPs clearly listed contributions from the three sectors (human health, veterinary health, agriculture). It was difficult to determine who was involved in the development of the South Africa NAP as a list of contributors was not available. Sierra Leone referred to human health and agriculture but did not refer to veterinary health specifically.

#### Context

The availability of antimicrobials, without prescriptions, was identified in Zimbabwe and Mauritius with self-medication reported in Ethiopia. Availability of antimicrobials from unskilled prescribers was possible in Nigeria. Students in Eritrea were found to share antibiotics and dispose of unwanted medications in the toilet. Within health care facilities, the use of antibiotics in surgery, including clean surgery, was common practice in Ethiopia, and inappropriate use was reported in the United Republic of Tanzania. Malawi reported that supporting AMR strategies was difficult with a lack of human resources and poor infrastructure with antibiotic prescriptions and dispensing regulated by the Pharmacy Board.

Inappropriate use of antimicrobials in animals was reported in Ghana, United Republic of Tanzania, Zimbabwe, Zambia and Eritrea where drugs and pesticides were used without a valid prescription, often at lower doses and for a shorter duration than recommended. This is partly attributed to value of livestock as the main source of income. The United Republic of Tanzania and Zambia identified farmers used antibiotics in livestock because of the lack of formal veterinary services and the prevalence of animal diseases. The lack of legislation in the animal health sector in Liberia is recognized as a gap in promoting responsible AMR.

Liberia identified poor infrastructure, inadequate supply chain management and the inclination for corruption in healthcare fund management as contributing to not implementing AMR strategies. Additional areas of concern included access to counterfeit antimicrobials through multiple borders that allow movement of humans, animals and goods.

SWOT analyses were provided by Eritrea, Ghana, Liberia, Malawi, Mauritius, Nigeria, United Republic of Tanzania while Eswatini, Ethiopia, Sierra Leone and Zambia. Zimbabwe provided a situation analysis. Kenya and South Africa did not provide a SWOT analysis but referred to a situation analysis developed earlier, which informed their NAP.

#### Content

The NAPs demonstrated good alignment with the strategic objectives presented in the WHO GAP. Eritrea was the only country to partially address Objective Five. Sierra Leone and South Africa included objectives to establish governance.

A number of issues contributing to countries being unable to progress with implementing AMR strategies were identified. The lack of legislation for the use of antimicrobials in raw materials of food and feed products in Eritrea was identified as a gap to progressing AMR strategies. The lack of infection prevention and control (IPC) programs, national animal vaccination problems and antimicrobial use in animals were identified as gaps in the United Republic of Tanzania. Zimbabwe and Mauritius reported the lack of dedicated IPC staff as a barrier to progression of AMR strategies. The lack of public awareness of AMR was identified in Nigeria and the United Republic of Tanzania. Economic challenges were limited improvements to infrastructure in Zimbabwe.

Liberia reported surveillance in place for tuberculosis, malaria and HIV resistance and is supported by the Esther Alliance for Global Health Partnerships in France. Liberia had the ability to perform MRSA surveillance and food borne disease antibiotic susceptibility. Zambia was enrolled in GLASS and performed national AMR surveillance for tuberculosis, malaria and HIV. Eritrea did not have a surveillance system but did have a national laboratory with capacity to detect AMR pathogens. Ghana reported paper-based national surveillance for tuberculosis and HIV. Ethiopia, Kenya, Mauritius, Nigeria, Sierra Leone, South Africa, United Republic of Tanzania and Zimbabwe did not have a national surveillance system. Zimbabwe highlighted constraints with laboratory testing facilities.

Eritrea, Ethiopia, Kenya, South Africa and the United Republic of Tanzania did not perform AST, while Zimbabwe mentioned AST as an action item. Liberia reported that they were able to identify priority organisms using the WHO Global Antimicrobial Resistance Surveillance System (GLASS). Mauritius reported they had a laboratory with AST capability and Sierra Leone reported their laboratories required reagents and human resources. Eswatini and Malawi did not discuss AST.

### Americas

#### Stakeholders and process

National action plans were written between 2017 (Barbados, Canada) and 2020 (USA). Two countries included all sectors (human, animal, agriculture), with USA not including veterinary health in the contributors list but discussing veterinary involvement throughout the NAP.

#### Context

Canada established IPC measures and standards in human and animal settings and found up to 50% of all antimicrobial prescriptions for humans were considered unnecessary. Canadian veterinarians can prescribe extra-label drug use because of the limited range of approved drugs for veterinary use. The USA identified mobility as an issue with more than 350 million travellers entering via more than 300 access points. No country (Barbados, Canada, United States of America) included a SWOT or situation analysis.

#### Content

All the NAPs demonstrated good alignment with the strategic objectives presented in the WHO GAP. Canada partially addressed Objective One. The USA acknowledged there were challenges to achieving every goal. Availability of AMR data from community and long-term care settings and antibiotic prescribing practices in hospital and all community settings were identified as gaps in Canada. Barbados did not provide specific gaps.

Both USA and Canada reported surveillance systems and AST were in place. The USA collaborated with the WHO GLASS while Canada used the Canadian Antimicrobial Resistance Surveillance System (CARSS), supported by public and private laboratories. The USA planned to enhance existing AST capability. Barbados did not have a surveillance system or AST.

### Eastern Mediterranean

#### Stakeholders and process

National action plans were written between 2016 (Iran) and 2020 (Oman, Palestine), with most written (n = 5) during 2017. Eleven of the 13 NAPs clearly listed contributions from the three sectors (human, animal, agriculture). It was difficult to determine who was involved in the Bahrain, Pakistan and Sudan NAPs as a list of contributors was not available. Iran did not specify the agriculture sector but listed Food and Drug Control. It was unclear if the veterinary sector contributed to the United Arab Emirates (UAE) NAP, however the sector was highlighted throughout the NAP. Oman did not include the veterinary sector as a contributor.

#### Context

Prescribing practices were identified as contributing to AMR. Antimicrobials can be purchased ‘over the counter’ (without a prescription) and used inappropriately in Egypt, Oman, Jordan, Palestine, and Kingdom of Saudi Arabia (KSA). Antimicrobials are used excessively for inpatients in KSA, and used inappropriately and freely available to livestock producers in Egypt, Iran, Iraq and Palestine. High rates of migration through medical tourism (Lebanon), religious events (KSA) or refugees (Jordan, Lebanon) contributed to poor AMR management. Ongoing conflict in Palestine was identified as a barrier to improving AMR management.

Egypt, Iran, Jordan, Lebanon, Pakistan, Palestine provided a SWOT analysis with Bahrain, Iraq, KSA, Libya, Sudan, UAE providing varying lengths of SWOT/situational analyses. Afghanistan, Oman did not provide specific contextual information.

#### Content

Nine of the NAPs demonstrated good alignment with the strategic objectives. All countries fully addressed Objective Two and Four. Bahrain was the only country to partially address Objective One. Objective Three was partially addressed by KSA, Oman, Palestine. Objective Five was partially addressed by Egypt, KSA, Libya, Oman. Afghanistan included an objective on national and international collaboration.

Lack of legislation and/or guidelines were identified in Palestine. The UAE highlighted the lack of national IPC standards for healthcare facilities and Jordan identified a lack of qualified AMR professionals. A lack of financial resources for AMR activities was identified in Egypt, Iraq, Jordan and Lebanon. Differing governance structures in human health care, such as public and private sectors with different legislation and funding made national coordination difficult in Iraq.

Five of the 14 countries did not have a national AMR surveillance system (Afghanistan, Bahrain, Iraq), Libya, Palestine. Some countries included the establishment or improvement of a national surveillance system as an objective. Three countries had a national surveillance system. Egypt was implementing surveillance in a phased approach, KSA identified they did not have coordination between health institutions, and Oman identified their system required an upgrade. Some countries were involved with or contributed data to GLASS (Iran, Lebanon, Palestine, Sudan, UAE) in the process of implementing this WHO surveillance system.

Antibiotic susceptibility testing, or steps toward it were reported in Egypt, Lebanon, Oman and Palestine. AST was not mentioned in 10 NAPs (Afghanistan, Bahrain, Iran, Jordan, KSA, Libya, Palestine, Sudan, UAE).

### Europe

#### Stakeholders and process

National action plans were written between 2009 (Czech Republic) and 2020 (Sweden) with most written (n = 5) during 2017. Nine of the 15 NAPs clearly listed contributions from the three sectors (human, animal, agriculture). It was difficult to determine who was involved in the Belgium, Netherlands, Republic of Serbia (RoS) and Former Yugoslav Republic of Macedonia (FYRoM) NAPs as a list of contributors was not available. Turkmenistan did not clearly include the agriculture sector as a contributor.

#### Context

Spain reported it is the fifth highest consumer of antibiotics in Europe. A high use of antimicrobials was identified in Serbia. Norway reported low use of antibiotics in humans and animals due to strategies such as the 99% reduction in antibiotic use in aquaculture due to vaccine use. Norway expressed concern regarding horizontal gene transfer with genetically modified organisms with resistance genes, leading to the development of restrictive policy. Sweden identified that growth promotants had been reduced in livestock with no loss of production. Good prescribing practices were identified as contributing to better practices in Ireland, Serbia, Sweden while a lack of formal supervision for prescriptions was identified in Turkmenistan.

Six countries provided a situational analysis (Finland, Ireland, Norway, RoS, Tajikistan, Turkmenistan). None of the remaining eleven countries provided a complete SWOT analysis (Belgium, Czech Republic, Denmark, France, Germany, Italy, Netherlands, Spain, Sweden, The FYRoM, United Kingdom (UK).

#### Content

Five of the NAPs demonstrated good alignment with the strategic objectives. The objectives were not addressed or could not be located for Belgium (full NAP was not written in English). Objective One was partially addressed by Czech Republic, Denmark, Italy, Netherlands, Norway, The FYRoM. Objective Two was partially addressed by Czech Republic, Denmark, France, Italy, Netherlands, Norway, The FYRoM. Objective Three was partially addressed by Czech Republic, Denmark, Italy, Netherlands, Norway, RoS, Spain, Turkmenistan and not addressed by Belgium or FYRoM. Objective Four was partially addressed by Czech Republic, Italy, Netherlands, Norway, FYRoM, Spain and not addressed by Belgium or Denmark. Objective Five was partially addressed by Denmark, Netherlands, Norway, RoS, Tajikistan, FYRoM and not addressed by Czech Republic, Italy, Turkmenistan. Germany included an objective including One Health and zoonoses and Sweden included an objective on leadership within the European Union and internationally.

A lack of training and AMR awareness were identified by Finland, France and Sweden. A complex health care delivery system (public and private) was highlighted by Ireland as contributing to gaps in AMR management.

Twelve of the 16 countries reported a range of AMR Surveillance Systems in use, with some including whether human and/or animal based (Belgium, France, Norway, RoS, Spain, UK). Some systems were country specific (Belgium, Denmark, Finland) while Germany used region specific systems and Ireland used WHO-GLASS. Czech Republic, FYRoM, Italy, Netherlands, Turkmenistan, Tajikistan reported they do not have AMR Surveillance in place. Sweden did not clearly identify their AMR Surveillance System.

Antibiotic susceptibility testing was available in Finland, Germany and Turkmenistan, underway in the UK and used with veterinary treatment failure (Spain). Spain stated they are improving reporting and aligning with existing programs such as EUCAST. AST was minimally or not discussed by Czech Republic, Denmark, FYRoM, Italy, Norway, RoS, Sweden, Tajikistan. The level of AST in Belgium, France, Ireland, Netherlands is unclear.

### Southeast Asia

#### Stakeholders and process

National action plans were written between 2015 (Thailand) and 2018 (DPR of Korea), with most written (n = 7) during 2017. Six of the nine NAPs clearly listed contributions from the three sectors (human, animal, agriculture). It was difficult to determine who was involved in the Maldives NAP as a list of contributors was not available. Bangladesh did not clearly include the veterinary sector and Democratic Republic of Timor Leste did not clearly include agriculture as contributors.

#### Context

Self-medication and easy access to antimicrobials was identified in Thailand, with Sri Lanka identifying greater rates of AMR when compared with the UK. Antimicrobial use for companion animals and livestock, as well as for agricultural crops, was identified by Sri Lanka and Thailand. Timor Leste identified limited capacity due to inadequate infrastructure and resources to respond to AMR.

Only Thailand provided a full SWOT analysis, with Bhutan, India, Indonesia, Maldives, and Democratic Republic of Timor Leste providing a situational analysis, with information ranging from comprehensive to limited. Bangladesh, DPR of Korea, Sri Lanka did not include a SWOT or situational analyses.

#### Content

Six of the NAPs demonstrated good alignment with all the strategic objectives. Objective One was partially addressed by Bhutan and not addressed by Bangladesh. Objective Two was partially addressed by Bangladesh and Bhutan. Objective Three was not addressed by Bangladesh and Bhutan. Objective Four was partially addressed by Bhutan and not addressed by Bangladesh. Objective Five was partially addressed by DPR Korea and not addressed by Bangladesh and Bhutan. The DPR of Korea included an objective for One Health.

The lack of a national policy was identified by Bhutan and DPR Korea with Thailand identifying resistance to strengthening AMR legislation. Limited human resources were identified in Thailand and Timor Leste with limited veterinary capacity for an increasing livestock population in the Maldives.

Two of the nine countries reported AMR Surveillance Systems in use. India and Sri Lanka both had a country specific system. Bangladesh, Bhutan, Democratic Republic of Timor Leste, DPR of Korea, Indonesia, Maldives reported they do not have an AMR Surveillance system in place. Thailand did not clearly identify their AMR Surveillance System.

Antibiotic susceptibility testing was available in Bhutan in three referral hospitals. Regional and Atoll hospitals in the Maldives provide some culture and sensitivity testing. It was minimally or not discussed by Bangladesh, Democratic Republic of Timor Leste, DPR of Korea, India, Indonesia, Sri Lanka, Thailand.

### Western Pacific

#### Stakeholders and process

National action plans were written between 2014 (Cambodia) and 2019 (Australia, Federated States of Micronesia, Japan, Lao People’s Democratic Republic (PDR), Nauru, Papua New Guinea (PNG), Republic of Marshall Islands) with Tuvalu written in 2021. Tuvalu and Nauru provided committee members roles but did not include names. Five of the nine NAPs listed contributions from the three sectors (human, animal, agriculture). It was difficult to determine who was involved in the NAPs from Australia, China, Mongolia, Philippines NAPs as a list of contributors was not available. The Republic of Marshall Islands did not include the veterinary and agriculture sectors as contributors.

#### Context

Inappropriate prescribing (Fiji) and use (Cambodia) of antimicrobials was identified in the respective NAPs, with Fiji reporting the use of some antimicrobials is synonymous with analgesia. Patient behaviours were identified as problematic in Japan, including patients changing dosage and duration of treatment, and self-prescribing for children by more than 33% of parents. The use of antimicrobials as growth promotants in production animals was identified in Cambodia and Lao PDR. Access of counterfeit antimicrobials were reported by Philippines. Antimicrobial residues had been found in animals consumed in Mongolia. Tuvalu does not have a trained veterinarian available.

Lao PDR provided a SWOT analysis, with Federated States of Micronesia, Fiji, Nauru, PNG, Republic of Marshall Islands, Tuvalu providing a situational analysis with information ranging from comprehensive to limited. None of the remaining six countries provided a SWOT or situational analysis (Australia, Cambodia, China, Japan, Mongolia, Philippines). Cambodia and Philippines both referred to a situational analysis being performed to guide the development of their NAPs.

#### Content

Seven of the NAPs demonstrated good alignment with all the strategic objectives. Objective One was partially addressed by Cambodia, China, Federated States of Micronesia, Republic of Marshall Islands. Objective Two was partially addressed by China and Federated States of Micronesia. Objective Three was partially addressed by Federated States of Micronesia and not by China. Objective Four was partially addressed by Cambodia and Federated States of Micronesia. Objective Five was partially addressed by Cambodia, China and Federated States of Micronesia. The Philippines included an objective for successfully becoming a national lead in AMR management. Japan included an objective for global policy development.

The lack of a national plan/strategy was identified by Cambodia. A dedicated AMR budget was flagged as necessary by Federated States of Micronesia. Lao PDR identified being landlocked and sharing borders with several countries a barrier to progressing with AMR plans. Lack of human resources were identified as needed in Papua New Guinea and Federated States of Micronesia, with Marshall Islands lacking a clinical pharmacist. Reduced access to resources such as disposable gloves was identified by Fiji. Both Nauru and Tuvalu are small countries with limited agricultural capability and reliance on livestock kept for food security.

Three countries reported AMR Surveillance Systems in use. Australia and Philippines both have a country specific system. Japan included AMR surveillance for veterinary practices. Mongolia reported they were about to enrol in WHO-GLASS. Lao PDR reported their surveillance system was not integrated between human, animal and agriculture sectors. Cambodia, China, Federated States of Micronesia, Fiji, Nauru, PNG, Republic of Marshall Islands, Tuvalu reported they do not have an AMR Surveillance system in place.

Antibiotic susceptibility testing was available in Australia with inconsistencies across laboratories. AST in Japan is used to identify specific pathogens such as drug resistant tuberculosis and used to improve the veterinary/agriculture sectors. One laboratory each in Fiji and PNG had some AST capacity. Tuvalu reported they did not have a microbiologist onsite. It was minimally or not discussed by Cambodia, China, Federated States of Micronesia, Lao PDR, Mongolia, Nauru, Philippines, Republic of Marshall Islands.

### National action plans not officially approved by the WHO

#### Stakeholders and process

The eight NAPs not officially approved by the WHO are from three (presumed) regions (Table 2). These plans were released between 2013 (Vietnam) and 2019 (Brunei), with three released in 2017, three in 2015 and two in 2016. Time periods for action were specified by six countries with Vietnam passing 2020 completion, Malaysia completing 2021, Myanmar 2022 and Brunei 2023.

#### Context

Availability of antibiotics without prescriptions occurred for people (Vietnam) and animals (Myanmar, Singapore). Poor sanitation and waste disposal from hospitals and manufacturing industries contribute to a risk of residues entering the environment such as drinking water. Existing legislation in Singapore prohibits the use of some antimicrobials in feed and food producing livestock and aquaculture. The availability of a single national electronic patient record was identified as a positive feature in Brunei.

No country conducted a SWOT analysis. Brunei, Malaysia, Myanmar, Switzerland, Vietnam, provided a situational analysis, ranging from comprehensive to limited information. Nepal, New Zealand, Singapore did not provide a situational analysis for their country.

#### Content

Five of the NAPs demonstrated good alignment with the strategic objectives. Objective One was partially addressed by Singapore, Switzerland, Vietnam. Objective Two was partially addressed by Singapore, Switzerland. Objective Three was partially addressed by Singapore, Switzerland, Vietnam. Objective Four was partially addressed by Singapore, Switzerland. Objective Five was partially addressed by Singapore, Switzerland.

A lack of data in research, antimicrobial usage or monitoring of aquaculture activities (Malaysia, Myanmar), between human and animal sectors (Nepal) or mechanisms of AMR in foodborne pathogens (Singapore) were all highlighted as essential information. Limited trained staff in Brunei contributed to adequate AMR implementation.

National AMR Surveillance systems are established/partially established in Malaysia, Nepal, New Zealand, and Singapore. Singapore identified gaps exist between integration of AMR testing and data sharing between the three sectors (human, animal, agriculture). Surveillance was predominantly in human health care facilities. New Zealand acknowledged that some surveillance and data was available for the three sectors (human, animal, agriculture). Countries that do not have any surveillance for AMR include Brunei, Myanmar, Switzerland, and Vietnam.

Antibiotic susceptibility testing varies greatly between countries. It was available in some hospitals (Malaysia), laboratories were identified as having basic capability (Myanmar). Improving reporting and aligning with existing programs such as EUCAST were noted (Singapore), while Switzerland complies with EUCAST already. Antibiotic susceptibility testing was mentioned as an action item (Nepal) or to improve capabilities within veterinary/agriculture (New Zealand) and not identified in Brunei, Vietnam.

## Discussion

Our review found that NAPs are diverse in their layout, actors, context and content. It is unclear what constitutes an official NAP for inclusion on the WHO NAP library. Plans range from five (Portugal) to 180 pages (UAE), with formats ranging from published documents ( e.g. UAE) to less formal documents (e.g. Netherlands). Both Malaysia and New Zealand published their NAPs in 2017 yet neither are available on the WHO NAP Library (as of 2 November 2021).

The WHO AMR Manual for developing NAPs (2016) and the WHO GAP (2015) discusses One Health and all sectors such as “human and veterinary medicine, agriculture, finance, environment, consumers”. We attempted to include the environment sector in our analysis. However, “Environment” is largely missing from the NAPs reviewed in this study. Indeed, stakeholders from other sectors such as finance, marine science, and community health, as examples, may provide a more balanced and practical perspective of how the NAP objectives can be achieved. The reduced level of engagement with veterinary and agriculture stakeholders with the development of the NAPs potentially reduces communication between all necessary sectors, which may impede One Health collaboration. Some countries do not have the capacity to include all three sectors, such as Nauru with no veterinarian and Republic of Marshall Islands with no pharmacist available. Displaying stakeholders provides transparency and may be seen as promoting One Health and collegiality between intersectoral stakeholders [2]. Participants involved with developing NAPs should be encouraged to include stakeholders from all three sectors (Human health, animal health, agriculture), and maintain regular progress meetings. Stakeholder involvement will differ between and within countries depending on priorities. Where livestock are the main income and contribute to food security, provision of veterinary care and land management advice are likely to contribute to more appropriate antimicrobial use.

There were considerable differences between available NAPs in how they were organized and the information they contained. NAPs that included the roles and positions of participants involved with the NAP development denoted a greater consultative process. However, the level of consultation is unknown and cannot be validated. The inclusion of a SWOT analysis provided a greater understanding of a countries ability to undertake activities to combat AMR successfully. Countries which provided useful and comprehensive SWOT analyses include, and are not limited to Eritrea, Mauritius and Nigeria (Africa). Situational analysis was provided by some countries. The quality of these varied and included some information relating to a country’s capability but were not as insightful as a SWOT analysis. Countries which did not include any SWOT or situation analysis made interpreting the level of capability difficult, as the information presented was very general, for example, Australia and New Zealand. The inclusion of a SWOT analysis can provide other countries to directly negotiate and provide assistance if they are in the position to do so, potentially contributing to reduced antimicrobial use.

Prohibiting factors to implementing AMR such as the use of freely available antimicrobials in livestock may appear to be related to lack of legislation or policy. Restriction of antimicrobials in livestock in easier to mandate in some countries rather than Eritrea or Bhutan where small stock holders are dependent on these animals for income and food [11, 12]. National policies in Singapore and Sweden prohibit antimicrobial use in livestock. Both of these countries also exhibit higher levels of food security [13, 14] so there may be less reliance on animals as a sole source of food or income for their family. The Sustainable Development Goals (food security) need to be considered for countries with greater dependence on livestock. Development of NAPs needs to consider the effects of restricting antimicrobial use with more viable and supportive options such as improving access to affordable veterinary care.

Achieving WHO GAP Objective 2 for surveillance and research is more challenging for low- and middle-income countries who lack infrastructure and qualified human resources. The establishment of surveillance for infectious diseases may be a result of prior programs such as the Joint United Nations Programme on AIDS [15]. Surveillance for AMR is difficult because it is less tangible and its effects are less easy to quantify at an individual and healthcare level [16].

The surveillance systems (such as GLASS [17], EUCAST [18]) in place in many countries around the world help to provide standardized data collection. The lack of data collection for animal health and agriculture is a feature of many countries that have submitted AMR NAPs [19]. Greater surveillance is necessary to have a true understanding of AMR from a One Health perspective.

### Factors contributing to enhanced national action plans

Most countries provided similar introductions of the impact of AMR globally or used information from another country (such USA HAI costs) rather than how AMR impacts on the country developing the NAP. This information would have been more meaningful with data related specifically to the country; for example, resistance levels of some antimicrobials, such as provided by Pakistan and Spain.

Some plans were limited in the information provided because of their brevity, for example, Belgium five pages, Czech Republic eight pages and Bangladesh 12 pages. The Netherlands had four additional documents available to support the Letter to Parliament in the original WHO database. These documents were not available in the updated database. The inclusion of these documents would have enhanced the information available.

Addressing the strategic objectives has been completed well by most countries. It is interesting that the thoroughness of the objectives is not related to the income level of the country. The inclusion of the information may be related to the information gained from the SWOT analysis or the skill of the WHO adviser and other stakeholders. The correlation between the strategic objectives and the inclusion of stakeholders, while weak may be an indication of the level of collaboration between sectors. Assumptions cannot be made from this finding but may be strengthened with examining any correlations between information included on the Tripartite AMR Country Self-Assessment survey rating progress on the strategic objectives.

There are distinct levels of achievement between countries. High income countries indicate they have progressed more in areas such as the establishment of National Surveillance systems. Low- and middle-income countries have more rudimentary requirements such as human and financial resources. Activities such as training or improving awareness amongst health professionals and the public were not clearly defined; it was thus difficult to measure and determine if achieved or not.

The Tripartite AMR Country Self-Assessment Survey (TrACSS) has been available since 2015 and allows all countries, whether they have a NAP or not, to contribute to global results of how they are combatting and contributing to AMR strategies. This survey provides a snapshot and progress report of what countries are developing, training and ongoing surveillance activities [20]. (Self-reporting introduces an element of bias and there is a reliance on the responder having a good understanding of English.) Responses to the GAP Strategic objectives would have supplemented the information gathered from the NAP but this activity was beyond the scope of the project.

### Limitations

A limitation of the study design is the inability to include the analysis of the 16 NAPs written in languages than English. More in-depth evaluation of each national action plan may enhance summarized information and provide a more comprehensive overview. Less than one third of countries in the world have provided a NAP precluding a complete evaluation of activities to combat AMR. The standard of submitted NAPs varies greatly and is likely to be influenced by the experience of the WHO regional advisor, other stakeholders or the capacity of the responsible person in the ministry. Despite the availability and clear instructions of the AMR Manual for developing NAPs, many countries do not appear to have referred to the manual during the development of their NAP.

Overall, the relationship of a country’s NAP objectives having good alignment with the WHO GAP strategic objectives is unclear. The diversity of NAPs does not allow accurate comparison. Further analysis is required to determine if NAP objectives alignment is an indication of a country’s existing activities or progress towards achieving targets to reduce AMR. Countries which have more easily identified needs are those that have provided SWOT or situational analyses. This may be an opportunity to globally promote these needs and allow other countries, which have capacity, to provide support. It may be prudent to evaluate the relationship between countries that are already supporting each other, such as Liberia and France or Malawi and United Kingdom. Countries which have not provided a NAP need to be contacted and offered practical assistance to help develop their NAP to combat AMR.

## Conclusion

The available NAPs for AMR provide a snapshot of objectives, activities underway and capabilities of countries around the world. It is unclear whether NAPs are describing existing activities underway or planned targets to be achieved during the designated time frame. A clear schedule of review, as depicted in the WHO NAP Guidelines would ensure NAPs could be standardized and more easily compared within and between regions. All countries should be encouraged to complete a SWOT analysis and include all relevant stakeholders (participants) involved in the process. Antimicrobial resistance is a dynamic and recognised One Health problem, which requires more regular reviews to assess the situation globally. The lack of acknowledgement and definition of One Health in the WHO Gap may inhibit other countries in adopting a One Health approach. Review and regular reporting with identification of barriers and enabling strategies may help other countries. It is vital to motivate and support the 12 non-WHO approved countries to get their NAP approved, as well as the 120 countries that have not submitted a NAP to date is required. Moreover, further exploration and explanation is important in countries with NAPs to identify the presence of the required structure and capacity to implement the plan.

## Supplementary Information

**Additional file 1**. Antimicrobial Resistance National Action Plans.

**Additional file 2**. Traffic light system for key National Action Plan variables (Green = fully addressed or located, Yellow = partially addressed or all responses not located, red = not addressed or cannot be located).

**Additional file 3**. Collaboration and stakeholder involvement/SWOT analysis and global alignment with WHO AMR GAP objectives WHO approved National Action Plans (n=70) and non-approved National Action Plans (n=8) (Note – not all names of countries visible in figure).

## Data Availability

The datasets analysed during the current study are available in the World Health Organization Library of AMR national action plans repository, https://www.who.int/teams/surveillance-prevention-control-AMR/national-action-plan-monitoring-evaluation/library-of-national-action-plans

## Abbreviations

AMR: Antimicrobial resistance
AST: Antibiotic susceptibility testing
CARSS: Canadian antimicrobial resistance surveillance system
DPR of Korea: Democratic People’s Republic of Korea
EUCAST: European committee on antimicrobial susceptibility testing
FYRoM: Former Yugoslav Republic of Macedonia
GLASS: Global antimicrobial resistance surveillance system
KSA: Kingdom of Saudi Arabia
Lao PDR: Lao People’s Democratic Republic
NAP: National action plan
PNG: Papua New Guinea
SWOT: Strengths, weaknesses, opportunities and threats
UAE: United Arab Emirates
UK: United Kingdom
USA: United States of Australia
WHO: World health organization
