# Supplementary material for: A review of national action plans on antimicrobial resistance: strengths and weaknesses
Source: Antimicrob Resist Infect Control. 2022 Jun 23;11:90. doi: 10.1186/s13756-022-01130-x (PMC9229779; doi:10.1186/s13756-022-01130-x)
Supplement: Supplementary file 1 — Additional file 1. Antimicrobial Resistance National Action Plans. [file 13756_2022_1130_MOESM1_ESM.docx]

Supplementary File 1 – Antimicrobial Resistance National Action Plans.

Available from World Health Organization Library of national action plans at https://www.who.int/teams/surveillance-prevention-control-AMR/national-action-plan-monitoring-evaluation/library-of-national-action-plans.

Afghanistan Ministry of Public Health. National Action Plan on Antimicrobial Resistance (NAP-AMR) 2017 – 2021. Afghanistan: Government of Afghanistan; 2017. p. 49.

Australian Government. Australia’s National Antimicrobial Resistance Strategy – 2020 and Beyond. Canberra, Australia: Commonwealth of Australia; 2020. Available from: https://www.amr.gov.au/resources/australias-national-antimicrobial-resistance-strategy-2020-and-beyond.

Bangladesh Ministry of Health and Family Welfare. National Action Plan: Antimicrobial resistance containment in Bangladesh (2017-2022). Bangladesh.: Bangladesh Ministry of Health and Family Welfare; 2017. p. 12.

Barbados Ministry of Health. Barbados National Action Plan on combatting Antimicrobial resistance 2017-2022. Barbados: Barbados Ministry of Health; 2017. p. 36.

Belgian Antibiotic Policy Coordination Committee. Policy Paper for the 2014-2019 term. Belgium: Belgian Government; 2014. p. 5.

Brunei Darussalam Ministry of Health and Ministry of Primary Resources and Tourism. Brunei Darussalam Antimicrobial resistance national action plan (2019-2023). Brunei: Brunei Government; 2018. p. 24.

Cambodian Ministry of Health. National Policy to combat Antimicrobial Resistance. Cambodia: Cambodian Government; 2014. p. 24.

Canadian Minister for Health. Tackling antimicrobial resistance and antimicrobial use: A Pan-Canadian Framework for Action. In: Minister for Health, editor. Canada: Canadian Government; 2017. p. 44.

Chinese Ministry of Health. National Action Plan to contain Antimicrobial Resistance. China: Chinese Ministry of Health; 2014. p. 16.

Costa Rica Ministry of Health. Costa Rica National Action Plan for Antimicrobial Resistance 2018-2025

Costa Rica: Costa Rica Ministry of Health; 2018.

Cyprus Ministry of Health. National Action Plan on Antimicrobial Resistance. Cyprus: Cyprus Ministry of Health; 2012. p. 50.

Czech Republic Ministry of Health. National Action Plan on Antimicrobial Resistance. Czech Republic: Czech Republic Government; 2011. p. 8.

Democratic People's Republic of Korea Ministry of Public Health. National Strategic Plan of Antimicrobial Resistance (2018-2020). DPR Korea: DPR Korea Ministry of Health; 2018. p. 60.

Democratic Socialist Republic of Sri Lanka Government. National Strategic Plan for Combating Antimicrobial Resistance in Sri Lanka (2017-2022). Sri Lanka: Democratic Socialist Republic of Sri Lanka Government; 2017. p. 55.

Denmark Ministry of Health. National Action Plan on Antibiotics in Human Healthcare: Three measurable goals for a reduction of antibiotic consumption towards 2020. Denmark: Denmark Ministry of Health; 2014. p. 24.

Egyptian Ministry of Health and Population. Egypt National Action Plan for Antimicrobial Resistance (2018-2022). Egypt: Egyptian Ministry of Health and Population; 2018. p. 66.

Eritrean Ministry of Health. Eritrean National Action Plan on Antimicrobial Resistance (2021-2025). Eritrea: Eritrean Government; 2021. p. 86.

Eswatini Ministries of Health and Agriculture and Natural Resource and Energy. Implementation Plan: National Antimicrobial Resistance Containment Strategic Plan (2018-2022). Eswatini: The Kingdom of Eswatini; 2020.

Ethiopian Food Medicine and Healthcare Administration and Control Authority. Strategy for the Prevention and Containment of Antimicrobial Resistance for Ethiopia. Ethiopia: Ethiopian Food Medicine and Healthcare Administration and Control Authority; 2015.

Federated States of Micronesia Department of Health and Social Affairs. National Multisectoral Action Plan on Antimicrobial Resistance for the Federated States of Micronesia (2019-2023). Micronesia: Federated States of Micronesia Department of Health and Social Affairs; 2019. p. 36.

Fiji Ministry of Health and Medical Services. Fiji National Antimicrobial Resistance Action Plan. Fiji: The Government of Fiji; 2015. p. 18.

Finland Ministry of Social Affairs and Health. National Action Plan on Antimicrobial Resistance (2017-2021). Finland: Finland Ministry of Social Affairs and Health; 2017. p. 60.

French Interministerial Committee on Health. Interministerial Roadmap for Controlling Antimicrobial Resistance. In: Interministerial Committee on Health, editor. France: French Interministerial Committee on Health, 2016. p. 38.

Ghana Ministries of Health Food and Agriculture and Environment and Technology and Innovation and Fisheries and Aquaculture Development. Ghana National Action Plan on Antimicrobial Resistance (2017-2021). Ghana; 2017. p. 112.

Government of India. National Action Plan ion Antimicrobial Resistance (2017-2021). India: Government of India; 2017. p. 57.

Government of Kenya. National Action Plan on Prevention and Containment of Antimicrobial Resistance (2017-2022). Kenya: Republic of Kenya; 2017. p. 52.

Government of Papua New Guinea. National Action Plan on Antimicrobial Resistance (AMR) (2019-2023). Papua New Guinea: Government of Papua New Guinea; 2019. p. 36.

Government of Sierra Leone. National Strategic Plan for Combating Antimicrobial Resistance (2018-2022). Sierra Leone, Government of Sierra Leone; 2018. p. 64.

Government of Switzerland. Strategy on Antibiotic Resistance Switzerland. Switzerland: Federal Council of Switzerland; 2015. p. 75.

Government of the Republic of Malawi. Antimicrobial Resistance Strategy (2017-2022). Republic of Malawi: Government of the Republic of Malawi; 2017. p. 128.

Government of the Republic of Zambia. Multi-sectoral National Action Plan on Antimicrobial Resistance (2017-2027). Republic of Zambia, Government of the Republic of Zambia; 2017. p. 79.

Government Offices of Sweden. Swedish Strategy to Combat Antibiotic Resistance (2020-2023). Sweden: Government Offices of Sweden; 2020. p. 24.

Her Majesty’s Government. Tackling antimicrobial resistance (2019-2024) The UK's five-year national action plan. United Kingdom: United Kingdom Government; 2019. p. 98.

Indonesian Ministry of Health. National Action Plan on Antimicrobial Resistance (2017-2019). Indonesia: Indonesian Ministry of Health; 2017. p. 57.

Iranian Ministry of Health and Medical Education. National Action Plan of the Islamic Republic of Iran for combating antimicrobial resistance (2016-2021). Iran: Iranian Ministry of Health and Medical Education; 2016. p. 120.

Iraqi Ministries of Health / Environment and Agriculture. National Action Plan of Antimicrobial Resistance in Iraq (2018-2022). Iraq: Government of Iraq; 2018. p. 50.

Ireland Department of Health. Ireland's National Action Plan on Antimicrobial Resistance (2017-2020). Ireland: The Ireland Department of Health; 2017. p. 114.

Italian Ministry of Health. National Action Plan on Antimicrobial Resistance (2017-2020). Italy: Italian Ministry of Health; 2017. p. 5, 80.

Jordanian Ministry of Health. National Action Plan for Combating Antimicrobial Resistance in the Hashemite Kingdom of Jordan. Jordan: Hashemite Kingdom of Jordan; 2018. p. 102.

Kingdom of Bahrain Ministry of Health. National Action Plan on Antimicrobial Resistance. Kingdom of Bahrain: Supreme Council of Health Kingdom of Bahrain; 2019. p. 50.

Kingdom of Saudi Arabia Ministry of Health. Kingdom Saudi Arabia national Action Plan on combating Antimicrobial Resistance. Kingdom of Saudi Arabia Kingdom of Saudi Arabia Ministry of Health; 2017. p. 36.

Lao PDR Ministries of Health and Agriculture and Forestry. National Strategic Plan on Antimicrobial Resistance in Lao PDR (2019-2023). Lao PDR: Lao PDR Ministries of Health and Agriculture and Forestry; 2019. p. 84.

Malaysian Ministries of Health and Agriculture and Argo-based Industry. Malaysian Action Plan on Antimicrobial Resistance (MyAP-AMR) (2017-2021). Malaysia: Ministry of Health Malaysia; 2017. p. 51.

Maldives Ministry of Health. National Action Plan for Containment of Antimicrobial Resistance (2017-2022). Maldives: Maldives Ministry of Health; 2017. p. 64.

Mongolian Minister of Health and Food and Agriculture and Light Industry. National Multi-sectorial Actin Plan on Combatting Antimicrobial Resistance (2017-2020). Mongolia: Mongolian Government; 2017. p. 18.

Myanmar Government. National Action Plan for Containment of Antimicrobial Resistance: Myanmar (2017-2022). Myanmar: Myanmar Government; 2017. p. 64.

Nepal Department of Health Services. National Antimicrobial Resistance Containment Action Plan Nepal. Nepal: Nepal Ministry of Health; 2016. p. 24.

Netherlands Minister of Health and Welfare and Sport. Approach to Antibiotic Resistance. Netherlands: Dutch House of Representatives; 2015. p. 15.

New Zealand Ministry of Health. New Zealand Antimicrobial Resistance Action Plan. Wellington New Zealand: Ministry of Health 2017. p. 29.

united stat

Nigerian Federal Ministries of Agriculture and Rural Development and Environment and Health. National Action Plan for Antimicrobial Resistance (2017-2022). Nigeria: Nigerian Ministries of Agriculture Environment and Health; 2017. p. 108.

Norwegian Ministries. National Strategy against Antibiotic Resistance (2015-2020). Norway: Norwegian Ministries; 2015. p. 36.

Oman Ministries of Health and Agriculture and Fisheries. Antimicrobial Resistance (AMR) National Action Plan. Oman: Sultanate of Oman; 2020. p. 32.

Pakistan Ministry of National Health Services. Antimicrobial Resistance National Action Plan Pakistan. Pakistan: Government of Pakistan; 2017. p. 64.

Philippine Department of Health. The Philippine Action Plan to Combat Antimicrobial Resistance: One Health Approach. Manila, Philippine: Department of Health; 2019. p. 40.

Republic of Lebanon Ministry of Public Health. National Action Plan on Combating Antimicrobial Resistance. Lebanon: Republic of Lebanon Ministry of Health; 2019. p. 149.

Republic of Liberia Ministry of Health. National Action Plan on Prevention and Containment of Antimicrobial resistance in Liberia (2018-2022). Liberia: Republic of Liberia Ministry of Health; 2017. p. 89.

Republic of Macedonia Ministry of Health. National Strategy for Containment of Antimicrobial Resistance (2012-2016). Republic of Macedonia Republic of Macedonia Ministry of Health; 2011. p. 18.

Republic of Marshall Islands Health and Human Services. National Multisectoral Plan on Antimicrobial Resistance for the Republic of Marshall Island (2019-2023). Republic of Marshall Islands Republic of Marshall Islands Health and Human Services; 2019. p. 21.

Republic of Mauritius Ministry of Health and Quality of Life. National Action Plan on Antimicrobial Resistance (2017-2021). Republic of Mauritius: Ministry of Health and Quality of Life; 2017. p. 36.

Republic of Nauru Government. National Multi-sectoral Plan on the Antimicrobial Resistance for the Republic of Nauru (2021-2025). Republic of Nauru Republic of Nauru Government; 2021. p. 24.

Republic of Serbia Ministry of Health. National Antibiotic Resistance Control Programmer for the period 2019-2021. Republic of Serbia Ministry of Health; 2019. p. 47.

Republic of South Africa Departments of Health and Agriculture and Forestry and Fisheries. South African Antimicrobial Resistance National Strategy Framework: A One Health Approach. Republic of South Africa Republic of South Africa Departments of Health and Agriculture and Forestry and Fisheries; 2018. p. 22.

Republic of Sudan Federal Ministry and Ministry of Animal Resources. National Action Plan on Antimicrobial Resistance (2018-2020). Republic of Sudan: Federal Ministry of Health; 2018. p. 53.

Republic of Tajikistan Ministries of Health and Social Protection and Agriculture. National Action Plan to Tackle Antimicrobial Resistance in the Republic of Tajikistan. Republic of Tajikistan Ministries of Health and Social Protection and Agriculture; 2018. p. 36.

Royal Government of Bhutan Ministry of Health. National Action Plan on Antimicrobial Resistance (2018-2022). Royal Government of Bhutan: Department of Medical Services; 2017. p. 29.

Singapore Agri-Food and Veterinary Authority and Ministry of Health and Natural Environment Agency and National Water Agency. National Strategic Action Plan on Antimicrobial Resistance. Singapore: Agri-Food and Veterinary Authority and Ministry of Health and Natural Environment Agency and National Water Agency; 2017. p. 24.

Spanish Government. National Action Plan for Antimicrobial Resistance (2019-2021). Spain: Spanish Government; 2019. p. 21.

State of Libya Ministry of Health. National Action Plan on Prevention and containment of Antimicrobial Resistance (2019-2023). State of Libya: Ministry of Health; 2018. p. 54.

State of Palestine Ministry of Health. National Action Plan for Antimicrobial Resistance (2020-2024). State of Palestine: Ministry of Health; 2020. p. 80.

Thailand Government. Thailand's National Strategic Plan on Antimicrobial Resistance (2017-2021). Thailand: Thailand Government; 2017. p. 66.

The German Federal Government. DART 2020: Fighting antibiotic resistance for the good of both humans and animals. Germany: Federal Ministry of Health; 2015. p. 32.

The Government of Japan. National Action Plan on Antimicrobial Resistance (2016-2020). Japan: The Government of Japan; 2016. p. 69.

The United Republic of Tanzania Ministry of Health Community Development Gender Elderly and Children. The National Action Plan on Antimicrobial Resistance (2017-2022). The United Republic of Tanzania: Ministry of Health Community Development Gender Elderly and Children; 2018. p. 76.

Timor-Leste Ministry of Health. National Action Plan on Antimicrobial Resistance: Timor Leste (2017-2020). Timor-Leste: Ministry of Health; 2017. p. 53.

Turkmenistan Ministry of Health and Medical Industry and State Sanitary and Epidemiological Service. National strategy for Containment of Antimicrobial Resistance in Turkmenistan (2017-2025). Ashgabat: Ministry of Health and Medical Industry and State Sanitary and Epidemiological Service; 2018. p. 40.

Tuvalu Ministry of Health. National Multisectoral Plan to Combat Antimicrobial Resistance (2021-2025). Tuvalu: Ministry of Health; 2021. p. 38.

United Arab Emirates Ministry of Health and Prevention. National strategy and Action Plan for Combatting Antimicrobial Resistance (NAP-AMR) (2019-2023). United Arab Emirates Ministry of Health and Prevention; 2019. p. 180.

United States of America Federal Task Force on Combating Antibiotic-Resistant Bacteria. National Action Plan for Combating Antibiotic-Resistant Bacteria (2020-2025). USA: Federal Task Force on Combating Antibiotic-Resistant Bacteria; 2020. p. 47.

Vietnam Ministry of Health. National Action Plan on Combatting Drug Resistance in the period form 2013-2020. Hanoi, Vietnam: Ministry of Health; 2013. p. 27.

Zimbabwe Government. Zimbabwe One Health Antimicrobial Resistance National Action Plan (2017-2021). Zimbabwe: Zimbabwe Government; 2017. p. 39.
