# Supplementary material for: A review of national action plans on antimicrobial resistance: strengths and weaknesses
Source: Antimicrob Resist Infect Control. 2022 Jun 23;11:90. doi: 10.1186/s13756-022-01130-x (PMC9229779; doi:10.1186/s13756-022-01130-x)
Supplement: Supplementary file 2 — Additional file 2. Traffic light system for key National Action Plan variables (Green = fully addressed or located, Yellow = partially addressed or all responses not located, red = not addressed or cannot be located). [file 13756_2022_1130_MOESM2_ESM.docx]

| Supplementary File 2 - Traffic light system for key National Action Plan variables.  (Green = fully addressed or located, Yellow = partially addressed or all responses not located , red = not addressed or cannot be located). | | | | | | | | | | | | |
| --- | --- | --- | --- | --- | --- | --- | --- | --- | --- | --- | --- | --- |
| **Region** | **Country** | **Multisectoral collaboration and involvement of stakeholders** | | | | **SWOT Analysis included** | **Alignment with WHO Global Action Plan on Antimicrobial Resistance Strategic Objectives** | | | | | **NAP Current 2021** |
|  |  | Human health | Veterinary health | Agriculture | Contributors listed |  | Objective 1 | Objective 2 | Objective 3 | Objective 4 | Objective 5 |  |
| Africa | Eritrea |  |  |  |  |  |  |  |  |  |  | 2025 |
|  | Eswatini |  |  |  |  |  |  |  |  |  |  | 2022 |
|  | Ethiopia |  |  |  |  |  |  |  |  |  |  | 2020 |
|  | Ghana |  |  |  |  |  |  |  |  |  |  | 2021 |
|  | Kenya |  |  |  |  |  |  |  |  |  |  | 2022 |
|  | Liberia |  |  |  |  |  |  |  |  |  |  | 2022 |
|  | Malawi |  |  |  |  |  |  |  |  |  |  | 2022 |
|  | Mauritius |  |  |  |  |  |  |  |  |  |  | 2021 |
|  | Nigeria |  |  |  |  |  |  |  |  |  |  | 2022 |
|  | Sierra Leone |  |  |  |  |  |  |  |  |  |  | 2022 |
|  | South Africa |  |  |  |  |  |  |  |  |  |  | 2024 |
|  | United Republic of Tanzania |  |  |  |  |  |  |  |  |  |  | 2022 |
|  | Zambia |  |  |  |  |  |  |  |  |  |  | 2027 |
|  | Zimbabwe |  |  |  |  |  |  |  |  |  |  | 2021 |
| Americas | Barbados |  |  |  |  |  |  |  |  |  |  | 2022 |
|  | Canada |  |  |  |  |  |  |  |  |  |  |  |
|  | USA |  |  |  |  |  |  |  |  |  |  | 2025 |
| Eastern Mediterranean | Afghanistan |  |  |  |  |  |  |  |  |  |  | 2021 |
|  | Bahrain |  |  |  |  |  |  |  |  |  |  | 2022 |
|  | Egypt |  |  |  |  |  |  |  |  |  |  | 2022 |
|  | Iran |  |  |  |  |  |  |  |  |  |  | 2021 |
|  | Iraq |  |  |  |  |  |  |  |  |  |  | 2022 |
|  | Jordan |  |  |  |  |  |  |  |  |  |  | 2022 |
|  | Kingdom of Saudi Arabia |  |  |  |  |  |  |  |  |  |  | 2018 |
|  | Lebanon |  |  |  |  |  |  |  |  |  |  | 2021 |
|  | Libya |  |  |  |  |  |  |  |  |  |  | 2023 |
|  | Oman |  |  |  |  |  |  |  |  |  |  | Not located |
|  | Pakistan |  |  |  |  |  |  |  |  |  |  | 2022 |
|  | Palestine |  |  |  |  |  |  |  |  |  |  | 2024 |
|  | Sudan |  |  |  |  |  |  |  |  |  |  | 2020 |
|  | United Arab Emirates |  |  |  |  |  |  |  |  |  |  | 2023 |
| Europe | Belgium |  |  |  |  |  |  |  |  |  |  | 2019 |
|  | Czech Republic |  |  |  |  |  |  |  |  |  |  | Not located |
|  | Denmark |  |  |  |  |  |  |  |  |  |  | Not located |
|  | Finland |  |  |  |  |  |  |  |  |  |  | 2021 |
|  | France |  |  |  |  |  |  |  |  |  |  | 2016 |
|  | Germany |  |  |  |  |  |  |  |  |  |  | 2020 |
|  | Ireland |  |  |  |  |  |  |  |  |  |  | 2020 |
|  | Italy |  |  |  |  |  |  |  |  |  |  | 2020 |
|  | Netherlands |  |  |  |  |  |  |  |  |  |  | 2019 |
|  | Norway |  |  |  |  |  |  |  |  |  |  | 2020 |
|  | Republic of Serbia |  |  |  |  |  |  |  |  |  |  | 2021 |
|  | Spain |  |  |  |  |  |  |  |  |  |  | 2021 |
|  | Sweden |  |  |  |  |  |  |  |  |  |  | 2023 |
|  | Tajikistan |  |  |  |  |  |  |  |  |  |  | Not located |
|  | The Former Yugoslav Republic of Macedonia |  |  |  |  |  |  |  |  |  |  | 2016 |
|  | Turkmenistan |  |  |  |  |  |  |  |  |  |  | 2025 |
|  | United Kingdom of Great Britain & Northern Ireland |  |  |  |  |  |  |  |  |  |  | 2024 |
| South-East Asia | Bangladesh |  |  |  |  |  |  |  |  |  |  | 2022 |
|  | Bhutan |  |  |  |  |  |  |  |  |  |  | 2022 |
|  | Democratic Republic of Timor Leste |  |  |  |  |  |  |  |  |  |  | 2020 |
|  | DPR of Korea |  |  |  |  |  |  |  |  |  |  | 2020 |
|  | India |  |  |  |  |  |  |  |  |  |  | 2021 |
|  | Indonesia |  |  |  |  |  |  |  |  |  |  | 2019 |
|  | Maldives |  |  |  |  |  |  |  |  |  |  | 2022 |
|  | Sri Lanka |  |  |  |  |  |  |  |  |  |  | 2022 |
|  | Thailand |  |  |  |  |  |  |  |  |  |  | 2021 |
| Western Pacific | Australia |  |  |  |  |  |  |  |  |  |  | 2020+ |
|  | Cambodia |  |  |  |  |  |  |  |  |  |  | 2017 |
|  | China |  |  |  |  |  |  |  |  |  |  | 2020 |
|  | Federated States of Micronesia |  |  |  |  |  |  |  |  |  |  | 2023 |
|  | Fiji |  |  |  |  |  |  |  |  |  |  | 2020 |
|  | Japan |  |  |  |  |  |  |  |  |  |  | 2020 |
|  | Lao PDR |  |  |  |  |  |  |  |  |  |  | 2023 |
|  | Mongolia |  |  |  |  |  |  |  |  |  |  | 2020 |
|  | Nauru |  |  |  |  |  |  |  |  |  |  | 2025 |
|  | Papua New Guinea |  |  |  |  |  |  |  |  |  |  | 2023 |
|  | Philippines |  |  |  |  |  |  |  |  |  |  | Not located |
|  | Republic of Marshall Islands |  |  |  |  |  |  |  |  |  |  | 2023 |
|  | Tuvalu |  |  |  |  |  |  |  |  |  |  | 2025 |
| National Action Plans not approved by WHO & included in original WHO NAP database | Brunei |  |  |  |  |  |  |  |  |  |  | 2023 |
|  | Malaysia |  |  |  |  |  |  |  |  |  |  | 2021 |
|  | Myanmar |  |  |  |  |  |  |  |  |  |  | 2022 |
|  | Nepal |  |  |  |  |  |  |  |  |  |  | 2025 |
|  | New Zealand |  |  |  |  |  |  |  |  |  |  | Not located |
|  | Singapore |  |  |  |  |  |  |  |  |  |  | Not located |
|  | Switzerland |  |  |  |  |  |  |  |  |  |  | 2020 |
|  | Vietnam |  |  |  |  |  |  |  |  |  |  | 2020 |
