# Supplementary material for: A review of national action plans on antimicrobial resistance: strengths and weaknesses
Source: Antimicrob Resist Infect Control. 2022 Jun 23;11:90. doi: 10.1186/s13756-022-01130-x (PMC9229779; doi:10.1186/s13756-022-01130-x)

Supplementary File 3 – Collaboration and stakeholder involvement/SWOT analysis and global alignment with WHO AMR GAP objectives WHO approved National Action Plans (n=70) and non-approved National Action Plans (n=8) (Note – not all names of countries visible in figure).

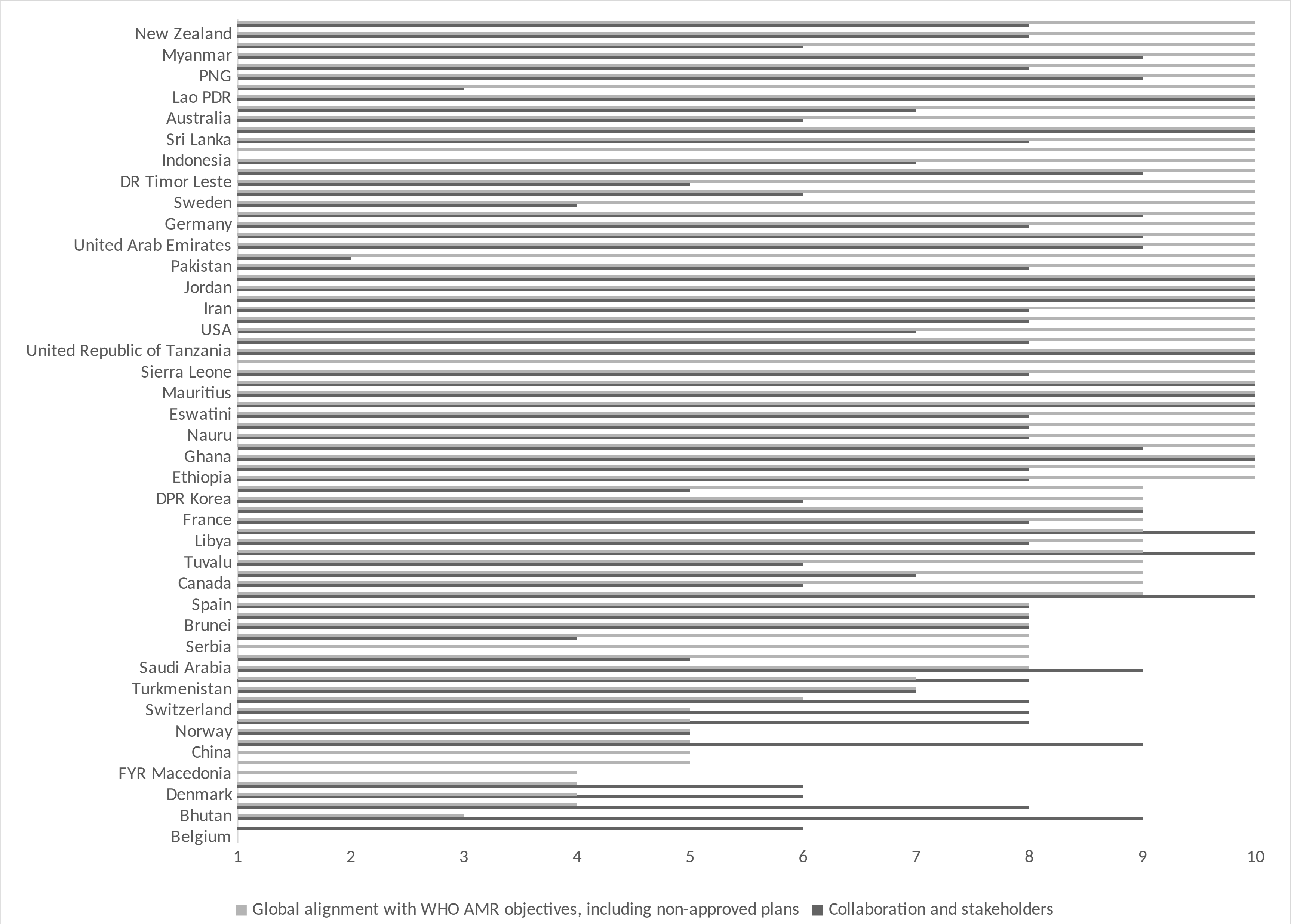

Supplement: Supplementary file 3 [file 13756_2022_1130_MOESM3_ESM.docx]
